# Class IIa Bacteriocins: Diversity and New Developments

**DOI:** 10.3390/ijms131216668

**Published:** 2012-12-06

**Authors:** Yanhua Cui, Chao Zhang, Yunfeng Wang, John Shi, Lanwei Zhang, Zhongqing Ding, Xiaojun Qu, Hongyu Cui

**Affiliations:** 1 School of Food Science and Engineering, Harbin Institute of Technology, Harbin 150090, China; E-Mails: yhcui@hit.edu.cn (Y.C.); zhangchao201089@163.com (C.Z.); dingzhongqing@hit.edu.cn (Z.D.); 2 State Key Laboratory of Veterinary Biotechnology, Harbin Veterinary Research Institute, Chinese Academy of Agricultural Sciences, Harbin 150001, China; E-Mail: gbhongyucui@126.com; 3 Guelph Food Research Center, Agriculture and Agri-Food Canada, Guelph, ON N1G5C9, Canada; E-Mail: john.shi@agr.gc.ca; 4 Institute of Microbiology, Heilongjiang Academy of Sciences, Harbin 150010, China; E-Mail: qvxiaojun@163.com

**Keywords:** class IIa bacteriocin, lactic acid bacteria, diversity, genetic organization, discovery

## Abstract

Class IIa bacteriocins are heat-stable, unmodified peptides with a conserved amino acids sequence YGNGV on their *N*-terminal domains, and have received much attention due to their generally recognized as safe (GRAS) status, their high biological activity, and their excellent heat stability. They are promising and attractive agents that could function as biopreservatives in the food industry. This review summarizes the new developments in the area of class IIa bacteriocins and aims to provide uptodate information that can be used in designing future research.

## 1. Introduction

Many Gram-positive bacteria, particularly many lactic acid bacteria (LAB) are known to secrete ribosomally-synthesized peptides or proteins that have antimicrobial activity. These compounds (bacteriocins) have been shown to display inhibitory activity against closely related bacteria [1,2]. Four classes of bacteriocins have been defined based on common characteristics, mainly primary structure, molecular weight, mode of action, heat stability and their genetic properties [1,2]. Among these classes, class II, consisting of small peptides that do not contain modified residues, has been divided further into subgroups. Class IIa bacteriocins are characterized by the occurrence of a highly conserved hydrophilic and charged *N*-terminal region that has a disulphide bond linkage [1,2]. In some bacteriocins, an additional disulphide bond is present. The unambiguous consensus amino acid sequence of class IIa bacteriocins is the “pediocin box” YGNGV (where V can be replaced by L in some cases) [1–3]. This consensus sequence is included in the conserved *N*-terminal region YGNGVxCxK/NxxC (where X is any amino acid) [1,2]. Class IIa bacteriocins show their strong inhibitory effect on *Listeria* sp. as well as other food spoilage and pathogenic bacteria. They have received much attention due to their generally recognized as safe (GRAS) status, their high biological activity, and their heat stability. These compounds show great promise and are attractive candidates for use as biopreservatives in the food industry [4–7].

## 2. Diversity of Class IIa Bacteriocins

To date, there are about 50 different kinds of class IIa bacteriocins that have been characterized to the extent that one can with a high degree of certainty determine whether the bacteriocin differs significantly from other bacteriocins (Supplementary Table 1). These bacteriocins have been isolated from a wide variety of LAB, including *Lactobacillus* sp., *Enterococcus* sp., *Pediococcus* sp., *Carnobacterium* sp., *Leuconostoc* sp., *Streptococcus* sp., as well as *Weissella* sp. [8,9]. They have also been found in the non-LAB *Bifidobacterium bifidum*[10,11], *Bifidobacterium infantis*[12], *Bacillus coagulans*[13] and *Listeria innocua*[14]. These bacteriocin-producing LAB have been isolated from various environments, including dairy products, fermented sausages, vegetables, and the mammalian gastrointestinal tract.

The class IIa bacteriocins are initially produced as a protein precursor containing an *N*-terminal leader peptide. This leader peptide is removed by site-specific proteolytic cleavage during export, to yield the mature bacteriocins [2,15]. These mature bacteriocins rang in length from 25 amino acids for mutacin F-59.1 to 58 amino acids for acidocin A. The classification of Gram-positive bacteriocins is complex and several authors have proposed different classifications based on different criteria [1–3,16–18]. The present direction for defining novel classification schemes of Gram-positive bacteriocins tends to take into account the composition, three-dimensional (3D) structure and mode of action of the bacteriocins. Classification of class IIa bacteriocins have been broadly defined first on the basis of their conserved *N*-terminal region, the “pediocin box,” and then subdivided into 4 subclasses through sequence alignments of the less conserved *C*-terminal region [3,17,19,20].

**Figure 1:**
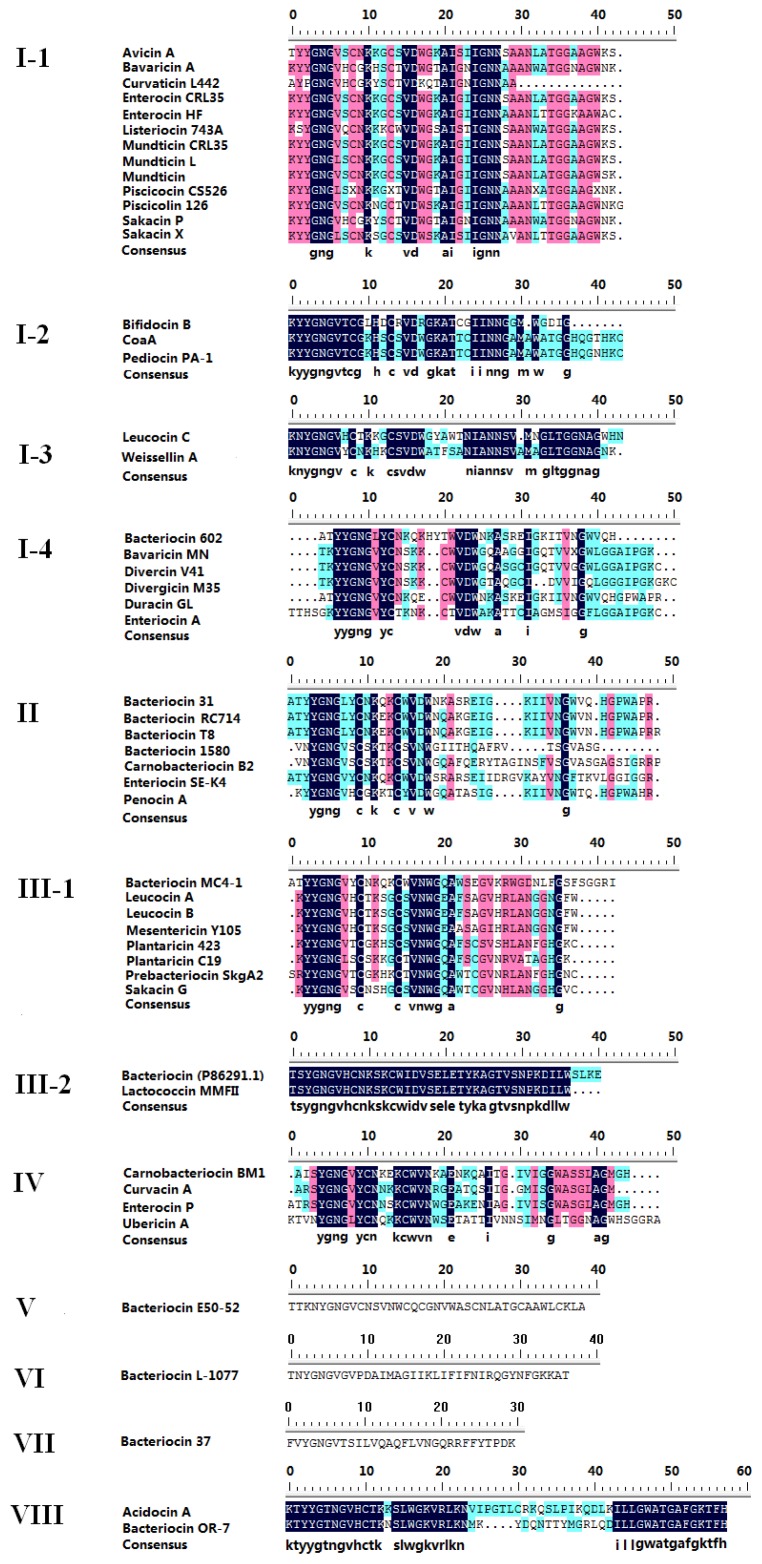
Multiple sequence alignment of class IIa bacteriocins.

The most recent repertoire of class IIa bacteriocins consists of 28 peptides [3]. In this paper, some class IIa bacteriocins were supplemented, including avicin A [21], bavaricin A [22], curvaticin L442 [23], enterocin CRL35 [24], enterocin HF (P86183), bifidocin B [10,11], ubericin A [8], weissellin A [25], bacteriocin 602 [26], bacteriocin 1580 [26], bacteriocin 37 [26], bavaricin MN [27], bacteriocin (P86291.1), bacteriocin E50-52 [28], acidocin A [29], bacteriocin OR-7 [30], bacteriocin L-1077 [31], mundticin L [32], leucocin B [33], prebacterioncin SkgA2, bacteriocin MC4-1 [34], and duracin GL. The 3D structures of bacteriocins were evaluated by SWISS-MODEL Workspace [35–37]. The 50 class IIa bacteriocins were classified into eight groups on the basis of their conserved primary structures, 3D structures and mode of action (See Figure 1). The results showed high consistency with the classification of class IIa bacteriocins that were described earlier and discussed by Nissen-Meyer *et al.*[3] (see Supplementary Table 1).

Group I contains 24 bacteriocins with a sequence length of between 25 and 49 amino acid residues. These peptides are secreted by 17 species of seven genera, including *Bacillus* sp., *Bifidobacterium* sp., *Carnobacterium* sp., *Enterococcus* sp., *Lactobacillus* sp., *Leuconostoc* sp., and *Weissella* sp. The bacteriocins in this group belong to subgroup 1 which was described in the classification of Nissen-Meyer *et al.*[3]. The bacteriocins of group I have a flexible hinge at the conserved Asp 17residue. This group can be further subdivided into three subgroups according to their sequence similarities and differences.

Subgroup I-1: includes avicin A, bavaricin A, curvaticin L442, enterocin CRL35, enterocin HF, listeriocin 743A, mundticin, mundticin CRL35, mundticin L, piscicocin CS526, piscicolin 126, sakacin P, and sakacin X. Members of this subgroup exhibit a common consensus motif IGNNxxANxxTGG located at the *C*-terminal region. Avicin A is produced by *Enterococcus avium* XA83 which was isolated from feces of healthy infants, and is a probiotic bacterium with diverse antimicrobial potential [21]. Mundticin L is virtually identical to enterocin CRL35. The only difference in sequence occurs in the fifth amino acid residue of the conserved sequence (YGNGX) of these mature bacteriocins, but this change has no influence on antimicrobial activity [32]. Sakacin P is produced by several *L. curvatus* strains LTH1174, L442 and CRL 705, which were isolated from Greek fermented sausages and fermented meat [38,39]; and by several *Lactobacillus sakei* strains I151 and LTH673 isolated from sausage and fermented meat [40,41].

Subgroup I-2 encompasses bifidocin B, coagulin, pediocin PA-1, which are produced by B. bifidum, B. coagulans, Enterococcus faecium, Lactobacillus plantarum, Pediococcus acidilactici, Pediococcus pentosaceus and Streptococcus mutans. The common consensus of this subgroup is KYYGNGVTCGK(L)HS(D)CS(R)VDW(R)GKATT(C)C(G)IINNG.

Pediocin PA-1/AcH is a 44-amino-acid class IIa bacteriocin produced primarily by strains of the genus *Pediococcus*, including *Pediococcus acidilactici* strains PAC1.0 [42], H [43,44], E, F, M [45,46], K10 [47], HA-6111-2, HA-5692-3 [48], MM33 [49]; *Pediococcus parvulus* ATO34, ATO77 [50] and *P. pentosaceus* FBB61 [51]. Pediocin PA-1/AcH is also synthesized by *L. plantarum* WHE92 [52], *L. plantarum* DDEN 11007 [53] and *E. faecium* Acr4.

The genetic determinants for the biosynthesis of pediocin PA-1/AcH are located within a plasmid-borne operon cassette in all producing lactic acid bacterial strains examined to date. In several strains, the sizes and organization of the various pediocin-encoding plasmids are similar [54–59]. It has been shown that the plasmids responsible for production in *P. acidilactici* H can be transferred intragenerically by conjugation [60]. The pediocin PA-1/AcH is the only class IIa bacteriocin for which both cross-species and cross-genera synthesis are known to occur [61].

The entire amino acid sequences of curvaticin L442 and bifidocin B have not been determined and the reported sequence for the bifidocin B contains some uncertainties. The mature sequence of enterocin CRL35 is identical to that of mundticin CRL35, but their leader sequences have some differences. The mature sequence of leucocin A was identical to that of leucocin B and they also had differences in their leader sequences. Sakacin P was identical to bavaricin A, and the peptide we list as sakacin P was a variant of sakacin P.

**Figure 2:**
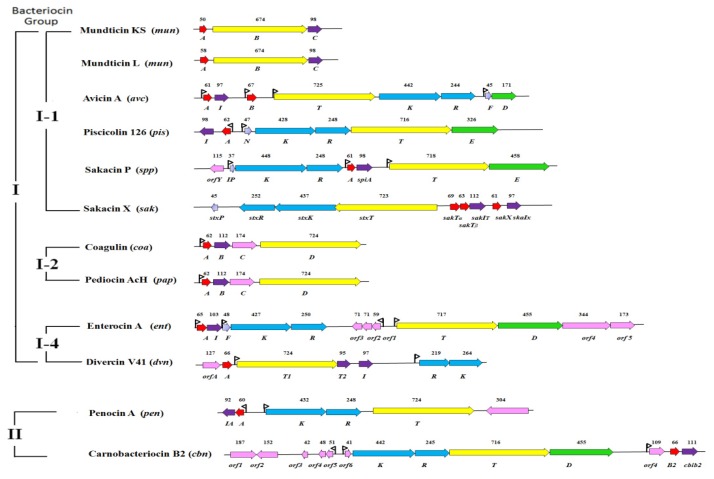

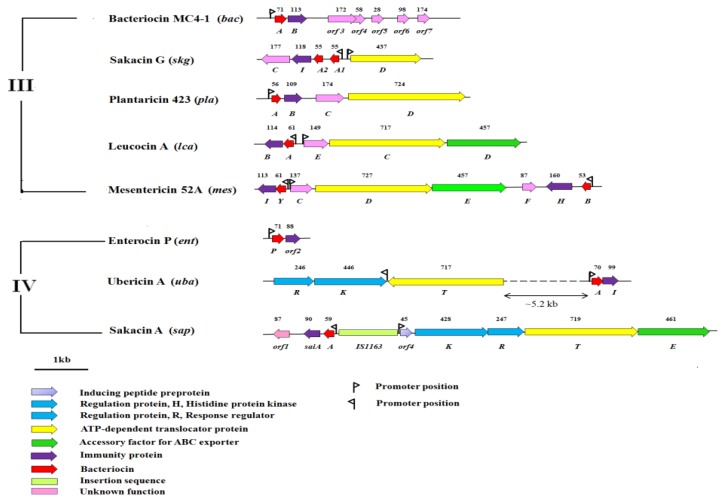
Organization of the gene clusters of class IIa bacteriocins. The figure was involved in production of avicin A in *Enterococcus avium* XA83 (*avc*, GenBank ID: FJ851402.1); bacteriocin MC4-1 in *Enterococcus faecalis* MC4 (*bac,* GenBank ID: EU047916.1); carnobacteriocin B2 in *Carnobacterium maltaromaticum* LV17B (*cbn*, GenBank ID: L47121.1); coagulin in *Bacillus coagulans* I_4_ (*coa*, GenBank ID: AF300457.1); divercin V41 in *Carnobacterium divergens* V41 (*dvn*, GenBank ID: AJ224003.1); enterocin A in *Leuconostoc gelidum* UAL 187 (*ent*, GenBank ID: AF099088); enterocin P in *Enterococcus faecium* P13 (*ent*, GenBank ID: AF005726.1); leucocin A in *Leuconostoc gelidum* UAL 187 (*lca*, GenBank ID: L40491.1); mesentericin 52A in *Leuconostoc mesenteroides* subsp. *mesenteroides* FR52 (*mes*, GenBank ID: AY286003.1); mundticin KS in *Enterococcus mundtii* NFRI 7393/AT06 (*mun*, GenBank ID: AB066267); mundticin L in *E. mundtii* CUGF08 (*mun*, GenBank ID: FJ899708.1); pediocin PA-1 in *E. faecium* Acr4 (*pap*, GenBank ID: HQ876214.1); penocin A in *Pediococcus pentosaceus* ATCC 25745 (*pen*, GenBank ID: NC_008525.1); piscicolin 126 in *Carnobacterium piscicola* JG126 (*pis*, GenBank ID: AF275938.1); plantaricin 423 in *Lactobacillus plantarum* 423 (*pla*, GenBank ID: AF304384); sakacin A in *Lactobacillus sakei* Lb706 (*sap*, GenBank ID: Z46867.1); sakacin G in *Lactobacillus sakei* CWBI-B1365 (*skg*, GenBank ID: EU570253.1) ; sakacin P in *Lactobacillus sakei* LTH673 (*spp*, GenBank ID: AF002276.1); sakacin X in *L. sakei* 5 (*sak*, GenBank ID: AAP44569.1); ubericin A in *Streptococcus uberis* E (*uba*, GenBank IDs: EF203953.1 and EF203954.1). Open reading frames (ORFs) encoding the related proteins are marked with the different color. The number of amino acid residues within each encoded protein is shown below the corresponding ORF.

Coagulin is produced by no-LAB *B. coagulan*s [13]. Interestingly, coagulin is almost identical to pediocin PA-1/AcH, showing 97.7% identity with pediocin PA-1/AcH. More specifically, the coagulin encoding DNA (*coaABCD* operon) showed 99% identity to that of the *papABCD* operon encoding the pediocin PA-1/AcH genes [62] (see Figure 2). A putative *mob-pre* (plasmid recombination enzyme) gene was identified in the coagulin-encoding plasmid pI_4_[13]. The *mob-pre* genes present on several plasmids extracted from various Gram-positive genera, including *Bacillus*, *Lactococcus*, *Streptococcus*, *Lactobacillus*, *Enterococcus*, and *Staphylococcus*[13]. In several cases, the corresponding *mob* genes have been shown to be required for conjugative mobilization and site-specific recombination [63]. Therefore, it was speculated that horizontal gene/operon transfer between *P. acidilactici* and *B. coagulans* was possible despite they being relatively unrelated, one is LAB, and the other is no-LAB [13,62].

Interestingly, mutacin F-59.1 from *Streptococcus mutans* 59.1 shared the conserved sequence KYYGNGVTCGKHSxSVDWxKXT [9]. *S. mutans* is a human indigenous oral bacterial species. It possesses an advantage against competitive species living in the same niche because of its bacteriocins [64]. The mutacin F-59.1 has a wide activity spectrum inhibiting human and food-borne pathogens [9]. Some amino acids of mutacin F-59.1 have not been determined.

In this subgroup, the bacteriocin-producing strains *B. bifidum* NCFB 1454 (bifidocin B) and *P. acidilactici* MM33 (pediocin PA-1), are from human intestinal origin [49,65]. They could be developed for their probiotic properties and as inhibitors of pathogenic bacteria in the gut. Pediocin PA-1 from *L. plantarum* DDEN 11007 and pediocin A from *P. pentosaceus* FBB61, are produced by bacteria with established probiotic properties [51,53,66].

Bifidocin B is the first class IIa bacteriocin from a member of the genus *Bifidobacterium*, sharing 56.8% homology with coagulin and inhibiting the growth of some species of the genera *Listeria*, *Bacillus*, *Enterococcus*, *Lactobacillus*, *Leuconostoc* and *Pediococcus*[11]. Recently, a new bacteriocin bifidin I from *Bifidobacterium* sp. was reported. Bifidin I from *B. infantis* BCRC 14602 and showed similarity with bifidocin B, but its whole sequences has not been determined [12]. Bifidin I showed a broad spectrum antimicrobial activity against Gram-positive bacteria and Gram-negative bacteria, including some food-borne pathogens, such as *Staphylococcus aureus*, *Bacillus subtilis*, *Bacillus cereus*, *Listeria monocytogenes*, *Clostridium butyricum*, *Salmonella enteritidis*, *Salmonella enterica* ssp., and *Shigella dysenteriae*[12].

Subgroup I-3 is represented by leucocin C, and weissellin A, which are produced by *Leuconostoc mesenteroides*, *Streptococcus uberis* and *Weissella paramesenteroides*. The common consensus of this subgroup is NYGNG(X)_2_C(X)_4_CXVXW(X)_6_IXNNS(X)_3_GLTG.

Leucocin C and leucocin C-TA33a are produced by different strains of *L. mesenteroides*, but they showed similar sequences [67]. Leucocin C-TA33a is from *L. mesenteroides* TA33a, which produced three bacteriocins (leucocin C-TA33a, leucocin B-TA33a and leucocin A-TA33a) with different inhibitory activity spectra [68,69]. The related research revealed that production of leucocin A-, B- and C-type bacteriocins was widespread in *Leuconostoc*/*Weissella* strains, including *Leuconostoc carnosum* LA54a, *W. paramesenteroides* LA7a, and *Leuconostoc gelidum* UAL 187-22 [68]. Weissellin A is a unique 4450 Da peptide which is produced by *W. paramesenteroides* DX which was isolated from a traditional Greek sausage. This bacteriocin exhibits strong activity against *L. monocytogenes*, *Listeria inocua* and *Clostridium sporogenes*[25].

Subgroup I-4 is represented by bacteriocin 602 [26], bavaricin MN [27], divercin V41, divergicin M35, duracin GL, enterocin A, which come from *Carnobacterium divergens*, *Enterococcus durans*, *E. faecium*, *L. sakei* and *Paenibacillus polymyxa*. The common consensus of this subgroup is YYGNGV(L)YC.

Group II contains bacteriocin 31, bacteriocin RC714, enterocin SE-K4, bacteriocin T8 (hiracin JM79), penocin A, bacteriocin 1580 and carnobacteriocin B2. The common consensus of this group is YGNGL(V)xCxKxxCxVxW. The bacteriocins in this group belong to subgroup 4 which was described in the classification of Nissen-Meyer *et al.*[3]. Most class II bacteriocin precursors contain a double-glycine-type signal peptide, and are translocated by dedicated ABC transporters and accessory proteins. However it is likely that some of these bacteriocins contain a different signal peptide. The sequence of hiracin JM79 is identical to that of bacteriocin T8. Hiracin JM79 is produced by *Enterococcus hirae* DCH5 isolated from wild mallard ducks, and contains a typical *sec* signal peptide that is believed to direct bacteriocins to the *sec* translocase embedded in the cytoplasmic membranes [70]. The bacteriocin 31, bacteriocin RC714 and enterocin SE-K4 are also *sec*-dependent class II bacteriocin [71,72].

Group III contains 10 bacteriocins, which can be further subdivided into two subgroups according to their sequence similarities and differences. The bacteriocins in this group belong to subgroup 2 which was described in the classification of Nissen-Meyer *et al.*[3].

Subgroup III-1, represented by 8 bacteriocins (bacteriocin MC4-1, leucocin A, leucocin B-Ta11a, mesentericin Y105, plantaricin 423, plantaricin C19, prebacteriocin SkgA2, and sakacin G) has a conserved N-terminal region YYGNGxxCxxxxCxVNWGxA. Plantaricin 423 is bactericidal for many Gram-positive food-borne pathogens and spoilage bacteria, including *Listeria* spp., *Staphylococcus* spp., *Pediococcus* spp., *Lactobacillus* spp. and so on [73]. Structurally, the *N* terminus of leucocin A (LeuA) consists of a three-strand antiparallel β-sheet (residues 2–16) that is rigidified by this (9-14)-disulfide moiety [74]. Bacteriocin MC4-1 and prebacteriocin SkgA2 are similar to leucocin A and leucocin A variant (C9L, C14L) in the 3D structures. There structures were determined by the SWISS-MODEL Workspace [35–37,75].

Subgroup III-2 consists of lactococcin MMFII and bacteriocin (P86291.1). Lactococcin MMFII is produced by *Lactococcus lactis* MMFII, which was isolated from a traditional Tunisian cheese [76]. Lactococcin MMFII is the first class IIa bacteriocin produced by a lactococcal strain. It has activity against closely related Gram-positive bacteria, including *Lactococcus lactis* subsp. *cremoris*, *Lactococcus lactis* subsp. *lactis*, *Lactobacillus delbrueckii*, *Lactobacillus casei*, *E. faecium*, *Enterococcus faecalis*, and *Listeria ivanovi*. The bacteriocin (P86291.1) is also produced by *Lactococcus* sp., showing 90.2% identity with lactococcin MMFII.

Group IV contains carnobacteriocin BM1, curvacin A, enterocin P and ubericin A. This group has the conserved sequences YGNGV(L)YCNxxKCWVNxxE. The group IV bacteriocins lack the hairpin-stabilizing tryptophan and/or cysteine residues that are present at or near the C-terminal end in most class IIa bacteriocins [3]. Carnobacteriocin BM1 is produced by *Carnobacterium piscicola* LV17B, which is isolated from fresh pork [77]. Curvacin A is produced by *Lactobacillus curvatus* LTH 1174, which originates from fermented sausage [78]. Enterocin P is produced by several *E. faecium* strains: IJ-31, P13, GM-1, ATB 197a, JCM5804T, LHICA 51, LHICA 28-4, and LHICA 40-4, which were isolated from various environments, such as fermented sausage, dairy products, feces of newborn infants, and non-fermented animal foods [79–84]. Enterocin P showed strong inhibitory action toward *Listeria* sp. It was processed and secreted by the sec-dependent pathway [79]. Ubericin A is the first streptococcal class IIa bacteriocin to be characterized [8]. It is composed of 49 amino acids with an YGNGL motif at the *N*-terminal half [8]. Although ubericin A showed high similarity with bacteriocins of subgroup I-3 in amino acid sequences, it showed high similarity with curvacin A in its 3D structure that was determined by SWISS-MODEL Workspace [35–37].

The bacteriocin E50-52, bacteriocin 37 and bacteriocin L-1077 are very different and form their own separate group. Bacteriocin E50-52 is produced by *E. faecium* NRRL B-30746, and shows diverse antimicrobial activity against both Gram-negative and Gram-positive bacteria, including *Campylobacter jejuni*, *Yersinia* spp., *Salmonella* spp., *Escherichia coli* O157:H7, *S. dysenteriae*, *Morganella morganii*, *Staphylococcus* spp., and *Listeria* spp. [28]. Bacteriocin 37 is produced by *P. polymyxa* NRRL B-30507, isolated from broiler chicken, and hasstrong antimicrobial activity against *C. jejuni*[26]. Bacteriocin L-1077 is produced by *Lactobacillus salivarius* 1077 (NRRL B-50053), isolated from poultry intestinal materials, and has broad-spectrum antimicrobial activity against 33 bacterial isolates (both Gram-negative and Gram-positive bacteria), including *L. monocytogenes* A 9-72, *E. coli* O157:H7, *Pseudomonas aeruginosa* 508 [31].

The group VII bacteriocins consists of acidocin A and bacteriocin OR-7. This group has a conserved *N*-terminal region KTYYGTNGVHCTKxSLWGKVRLKN and conserved *C*-terminal region ILLGWATGAFGKTFH. Acidocin A is produced by *L. acidophilus* with activity against *L. monocytogenes* and other closely related Gram-positive bacteria [29]. Bacteriocin OR-7 has 65.5% amino acids sequence similarity with acidocin A with a *C*-terminal region that is 100% identical to that of acidocin A. Interestingly, bacteriocin OR-7 has different antimicrobial activity from acidocin A. It is active against both Gram-negative and Gram-positive bacteria [30] and has strong antimicrobial activity to Gram-negative bacterium *C. jejuni* in the chicken gastrointestinal system [30].

The bacteriocin OR-7 and acidocin A have some differences with other class IIa bacteriocins. As a result there is a divergence of opinion as to whether bacteriocin OR-7 and acidocin A should be placed in the class IIa family of bacteriocin [3,19,29,30]. The position of the second cysteine is very different from the very conserved position of this cysteine in the class IIa bacteriocins, suggesting that bacteriocin OR-7 and acidocin A have a different 3D structure in their *N*-terminal region than the well conserved 3-stranded antiparallel β-sheet like structure which seems to be conserved in most class IIa bacteriocins [3]. Moreover, the sequence and length of the *C*-terminal region of bacteriocin OR-7 and acidocin A are also very different from other class IIa bacteriocins.

Both bacteriocin OR-7 and acidocin A contained a “pediocin box”-like motif, YGNGVXCXnV, in the *N*-terminal region of the peptide typical of class IIa bacteriocins, except that a T was present as YGTNGV in the sequence [29,30]. Based on our assessment of previous studies, we are in agreement that bacteriocin OR-7 and acidocin A belong to class IIa family [19,29,30].

## 3. Biosynthesis of Class IIa Bacteriocins

At least four genes are required for the production of class IIa bacteriocins, including a bacteriocin structural gene encoding a precursor, an immunity gene encoding an immunity protein, genes encoding an ATP-binding cassette transporter and an accessory protein for extracellular translocation of bacteriocin [2].

The class IIa bacteriocin production was regulated by quorum sensing (QS) system. QS systems are present in the majority of Gram-positive and Gram-negative bacteria, as one primary mechanism for bacteria to monitor the environment for other bacteria and to alter behavior on a population-wide scale in response to changes in the number and/or species present in a community [85–87].

QS systems used for the regulation of class IIa bacteriocin production are composed of three gene products, including an inducer peptide, a membrane-associated histidine protein kinase (HPK), and a cytoplasmic response regulator (RR) [88]. The inducer peptide is ribosomally synthesized at low levels as a precursor which appears not to be biologically active and contain an *N*-terminal extension or leader sequence [89]. Subsequent cleavage of the precursor at a specific processing site removes the leader sequence from the antimicrobial molecule concomitantly. Then inducer peptide is secreted and exported through the dedicated transport system involving an ABC-type translocator and an accessory protein [15,88,89]. The presequence of the bacteriocin plays a dual role in bacteriocin biosynthesis [2]. One is a protective role at the cytosolic side of the cell membrane by keeping the bacteriocin inactive. The other is as a recognition signal during export [2].

At a certain concentration threshold of the externalized inducer peptide, the transmembrane HPK detects a change in environmental signal and is activated, leading to its autophosphorylation [88,90]. Then the phosphorylated HPK transfers a phosphate group to its cognate RR. The phosphorylated RR acts as a transcriptional activator and activates expression of bacteriocin-related genes, including genes encoding bacteriocin, immunity protein, secretory apparatus, and regulatory proteins [2,88]. Bacteriocin and immunity genes most often reside on the same operon and are expressed concomitantly. The bacteriocin producer cells protect themselves from their own bacteriocin by the immunity protein. At a certain time, essentially all bacteriocin producer cells in the population are believed to secrete bacteriocins, and this result in a rapid activation of the bacteriocin production [89].

## 4. Genetic Organization of DNA Coding for Class IIa Bacteriocins

Generally, most class IIa bacteriocin genes are arranged in one or a few operons, which include a bacteriocin structural gene encoding a precursor, an immunity gene encoding an immunity protein, genes encoding an ATP-binding cassette transporter and an accessory protein for extracellular translocation of bacteriocin, and in several cases two regulatory genes encoding a two component system for regulations of the biosynthesis of bacteriocin [19] (Figure 2).

Production of bacteriocins is often correlated with the presence of a plasmid. Several class IIa bacteriocins, for example, enterocin A, divercin V41, sakacin P, carnobacteriocin B2 and carnobacteriocin BM1, have genes that have been shown to be located on chromosome fragments [19,77,91–93]. In many bacteriocin-producing bacteria, the bacteriocin structural gene and other related genes were located in one operon. However, genes encoding immunity and secretion functions may not always be linked to structure genes [89,94].

At the present time, all known class IIa bacteriocins are ribosomally synthesized as precursor peptides with an N-terminal leader sequence. The leader sequences of most bacteriocins contain two conserved glycine residues, which may serve as a recognition signal for protein processing and secretion. This double-glycine-type leader sequences were cleaved and removed by ATP-binding cassette (ABC) transporters and their accessory proteins [2]. However, a few class IIa bacteriocins, including bacteriocin 31, enterocin P, enterocin SE-K4, listeriocin 743A, and hiracin JM79 are secreted by the general *sec*-dependent export system [14,70–72,79,95]. These bacteriocins have a hydrophobic *N*-terminal *sec-*dependent leader sequence, which directs the secretory protein to the cytoplasmic membrane and is processed by a signal peptidase during translocation across the cytoplasmic membrane. The related genes for production of these bacteriocins are unknown [14,71,72,79,95–98].

Class IIa bacteriocins show a remarkable conservation of gene arrangement (Figure 2). The genetic organization of leucocin A gene cluster (*lca* locus) from *L. gelidum* UAL187 is a typical bacteriocin locus [99]. The *lca* locus includes two different directions operons with four bacteriocin-related genes *lcaA*, *lcaB*, *lcaC* and *lcaD*. The immunity protein gene *lcaB* is located immediately downstream of the structural leucocin A gene *lcaA*. The accessory transporter gene *lcaD* occurs also downstream of gene *lcaC* encoding an ABC transporter [99].

The genetic organization of sakacin P gene cluster (*spp* locus) from *L. sakei* LTH673 and LTH674 is complicate, when compared to leuconcin A [40,93]. It is composed of three operons, which encode a 61-amino-acid sakacin P precursor SppA, a sakacin P immunity protein SpiA; a transport and secretory system (a 718-amino-acid ABC transporter protein SppT and an accessory factor for ABC transporter protein SppE); and a three-component regulatory system (inducing peptide preprotein SppIP, HPK SppK and RR SppR), respectively [40,93]. The production of sakacin P in *L. sakei* Lb674 and LTH673 is regulated by a typical peptide pheromone-based QS mechanism [40,93].

The genetic organization of divercin V41 presents an unusual organization [92]. The *dvn* locus encodes a 66-amino-acid divercin V41 precursor, an ATP dependent transporter, two immunity-like proteins and two components of a lantibiotic-type signal-transducing system [92] (see Figure 2). Interestingly, a so-called transport accessory protein was absent from the locus. Generally, the genes encoding the HPK are located upstream of the genes encoding RR in anti-listeria bacteriocin operon [100]. However, in the *dvn* locus of divercin V41, the HPK gene followed the RR gene, which is a characteristic of lantibiotic operons. The genetic organization of the fragment suggests important gene rearrangements [92].

Sometimes one locus can include productions of two bacteriocins. *L. sakei* 5 produces a plasmid-encoded bacteriocin sakacin P, as well as two chromosomally encoded bacteriocins, *i.e.*, sakacin T, which is a class IIb two-peptide bacteriocin and sakacin X, which is a class IIa bacteriocin [101]. The sakacin TX locus encodes structural genes of sakacin T and sakacin X, including two adjacent but divergently oriented gene clusters (See Figure 2). The first gene cluster *stxPRKT* is believed to encode an inducing peptide, three proteins involved in regulation and secretion of these bacteriocins. The second gene cluster includes *sakT_α_*, *sakT_β_*, *sakI_T_*, *sak_X_* and *sakI_X_*, which encode the structural and immunity genes for sakacin T and sakacin X [101].

*L. mesenteroides* FR52 produces both mesentericin 52A and 52B [102]. Mesentericin 52A is a 37-amino-acid class IIa bacteriocin, identical to mesentericin Y105 from *L. mesenteroides* Y105 [103]. Mesentericin 52B is a 32-amino-acid atypical class II bacteriocin, identical to mesentericin B105 from *L. mesenteroides* Y105 [104]. The *mes* locus of *L. mesenteroides* FR52 is involved in productions of mesentericin 52A and 52B [104]. The previous study revealed that ATP dependent transporter MesD and transport accessory protein MesE were involved in secretion and transport of these bacteriocins [104]. Mesentericin 52A and mesentericin 52B have own immunity genes *mesI* and *mesH*, respectively.

The sakacin G gene cluster (*skg* locus) from *L. sake* 2512, R1333 and CWBI-B1365 was very interesting because it contained duplicated structural genes *skgA1* and *skgA2*[105–107]. There is only a two-amino-acid difference in sequence occurs in leader peptides of these prebacteriocins which makes these mature peptides, SkgA1 and SkgA2, essentially identical [106,107].

The genetic organization of avicin A gene cluster (*avc* locus) from *E. avium* has been established [21]. It is the first bacteriocin locus identified in *E. avium* to be characterized at the molecular level [21]. The locus showed a particular gene organization. The accessory gene *avcD* associated with bacteriocin transport did not occur immediately downstream of the gene *avcT* (which encodes an ABC transporter), but two regulatory genes *avcK* (which encodes a HPK) and *avcR* (which encodes a RR) followed the gene *avcT*[21]. The *avcK*, *avcR*, and induction peptide pheromone-encoding gene *avcF*, constituted a three-component regulatory system in the avicin locus. This indicated that the production of avicin A was regulated by the peptide pheromone-inducible regulatory system [21]. For most class IIa bacteriocins, three genes responsible for regulation are located in the same operon, but *avcK*, *avcR*, and *avcF* were located in two different operons (See Figure 2). In this locus includes two bacteriocins structural genes *avcA* and *avcB*. Avicin B is a divergincin-like bacteriocin, but it didn’t show antimicrobial activity and is probably a relic of a previous functional bacteriocin [21].

## 5. Structure-Function Relationship and Target Recognition of Class IIa Bacteriocins

To date, the 3D structures of leucocin A [74], carnobacteriocin B2 [108], sakacin P [109] and curvacin A [110] have been characterized by nuclear magnetic resonance (NMR) spectroscopy. The 3D analysis revealed that class IIa bacteriocins consist of a hydrophilic, cationic and highly conserved *N*-terminal β-sheet domain, and a flexible, diverse hydrophobic/amphiphilic *C*-terminal domain [3,74,108–110]. The former is structurally stabilized by a conserved disulfide bridge; the latter contains a central amphiphilic α-helix, ending with a structurally extended *C*-terminal tail. The amphipathic α-helix was critical for antimicrobial specificity and temperature-dependent activity of these class IIa bacteriocins [74,108,111–114]. The *C*-terminal part of some class IIa bacteriocins, such as enterocin A, divergicin M35, divercin V41, coagulin, pediocin PA-1, sakacin G and plantaricin 423, formed a hairpin structure which was stabilized by a disulfide bridge between a cysteine residue in the middle of the α-helix and a cysteine residue at the *C*-terminus [3].

Two cysteines that come from the conserved *N*-terminal region (YGNGVxCxK/NxxC) of class IIa bacteriocins formed a conserved disulfide bond. In most class IIa bacteriocins, the disulfide bond is formed between cysteine^9^ and cysteine^14^. Extensive studies indicate that this conserved disulfide bond is required for antimicrobial activity for class IIa bacteriocins [115–117]. Mutants of mesentericin Y105 (cysteine^9^→serine^9^, cysteine^14^→serine^14^) showed a marked loss in antimicrobial effects [115]. The antimicrobial activity of pediocin PA-1 was abrogated by the substitution of 11 different amino acids at cysteine^14^ based on NNK scanning [116]. Substitution of the cysteines with serines in leucocin A (LeuA) abolished antimicrobial effects [117].

However, some results from Derksen *et al.* indicated that the disulfide bond in leucocin A (LeuA) could be replaced by a noncyclic diallyl moiety without significant loss in activity [117]. The leucocin A (C9F, C14F), bis-allyglycine-leucocin A, and norvaline-leucocin A retained activities comparable to that of the natural leucocin A [75,114]. The researchers speculated that hydrophobic or π-stacking interactions can compensate for the absence of the disulfide in this molecule and assist receptor binding [75,114,117].

Three analogues of leucocin A (LeuA) and six analogues of pediocin PA-1(Ped) were synthesized by replacing the conserved cysteines that form a disulfide bond with pairs of hydrophobic amino acids [114]. Noncovalent hydrophobic interactions in all of the leucocin A (LeuA) derivatives effectively replaced the disulfide and afforded peptides with full antimicrobial activity [114]. Apparently the propensity of the intraloop sequence of leucocin A (LeuA) to induce β-turns in combination with the hydrophobic interaction of the two Phe residues is sufficient to achieve the appropriate conformation for bioactivity [114,118].

Sit *et al.* presented the 3D solution structures of the inactive (C9S, C14S)-leucocin A and the active (C9L, C14L)-leucocin A peptides [75]. Mutation of the two cysteine residues to serines or leucines did not affect the overall charge of the peptide, and therefore is highly unlikely to interfere with the electrostatic interactionsbetween the peptide and the bacterial cell surfaces. It was speculated that the N terminus may be serving a more crucial function, such as forming intermolecular contacts with other leucocin A–EII_t_^man^ complexes during pore formation [75].

Receptor binding might occur on the surface of a three-strand antiparallel β-sheet at the *N* terminus of the peptide as well as by recognition of the hydrophobic face of the amphipathic *C*-terminal α-helix, which is known to be required and determines specificity for particular organisms [112,119,120]. These results indicate that although the *N*-terminal loop has a vital influence on the activity of the peptide, additional interactions at the *C* terminus with the receptor must match and contribute to the overall activity [115,119–121].

Most class IIa bacteriocins present a single intramolecular disulfide bond between cysteine^9^ and cysteine^14^. The *C*-terminal part of a few class IIa bacteriocins, contains an additional *C*-terminal disulfide bridge, such as sakacin G (between cysteine^24^ and cysteine^37^), plantaricin 423 (between cysteine^24^ and cysteine^37^), pediocin PA-1/AcH (between cysteine^24^ and cysteine^44^), divercin V41 (between cysteine^25^ and cysteine^43^), and enterocin A (between cysteine^29^ and cysteine^47^). The second disulfide bridge not only plays an important role in stabilizing the 3D structure of the *C*-terminal domain, but also correlates strongly with spectrum of activity [2,20,109,113,122,123]. The previous studies indicated that the second disulfide bridge in the class IIabacteriocins contributes to widening of the antimicrobial spectrum as well as to higher potency at elevated temperatures [113].

It is well known that class IIa bacteriocins kill target cells by forming pores and disrupting the integrity of target cell membranes, causing dissipation of proton motive force, depletion of interacellular ATP and leakage of amino acids and ions [2,19]. Numerous mode-of–action studies have demonstrated that the sugar transporter mannose phosphotransferase system (Man-PTS) serve as target receptors for class IIa bacteriocins on sensitive cells [124–131]. The Man-PTS, which is a complex sugar uptake system in the Gram-positive *Firmicutes* and Gram-negative *Gammaproteobacteria*, includes a general PTS protein enzyme I (EI), a histidine containing phosphocarrier protein (HPr) and a carbohydrate-specific protein complex (enzyme II, EII) [132].

The enzyme II consists of four subunits: IIA, IIB, IIC and IID [132]. Subunits IIA and IIB are located in the cytoplasm and are responsible for phosphorylation. They are often found together on one protein. The IIC subunit is an integral membrane protein involved in sugar transport. The IID subunit is also a transmembrane protein [132]. The membrane proteins IIC and IID together form a membrane-located complex. IIA and IIB are in reversible contact with the membrane-located complex [129,133]. Other studies indicated that a single extracellular loop of the membrane-located protein IIC (MptC) was involved in specific target recognition by the class IIa bacteriocins, and was the major determinant responsible for species-specificity [125,130].

The proposed mechanism of action for IIa bacteriocins is as follows: first, the *N*-terminal β-sheet domain of bacteriocin binds to the extracellular loop of IIC in the Man-PTS. Then, *C*-terminal α-helix-containing hairpin or hairpin-like domain of the bacteriocin interacts with the transmembrane helices of the Man-PTS, leading to conformational changes in the Man-PTS proteins in a manner that renders the transporter irreversibly open thereby causing uncontrolled efflux of essential molecules, disruption of the membrane integrity and in effect, cell death [131,134]. In bacteriocin producing cells, a cognate immunity protein tightly binds the receptor in a bacteriocin-dependent manner, to prevent killing by the bacteriocin [129]. However some class IIa bacteriocins, including enterocin P and sakacin A, showed a different mode of receptor recognition. They employ the IIC and IID complex as a receptor on target cells and then the cognate immunity protein (LciA) is tightly associated with the bacteriocin-receptor complex to render producer cells immune [129,135].

Most class IIa bacteriocins have a relatively narrow inhibitory spectrum, inhibiting predominantly genera or species closely related to the bacteriocin producers. In order to reveal the mechanism of the receptor function specificity, a phylogenetic analysis of membrane-located proteins (IIC and IID) of 86 Man-PTSs from a wide range of bacterial genera was performed [136]. These man-PTSs are clustered into three distinct groups, named groups I, II and III. Fourteen man-PTSs distributed all over the phylogenetic tree were selected for heterologous expression in *L. lactis* indigenous man-PTS-deletion mutant [136]. Bacteriocin sensitivity of the different *L. lactis* clones was determined with four class IIa bacteriocins, including pediocin PA-1, enterocin P, sakacin P, and penocin A [136]. The results indicated that only members of group I could serve as receptors for class IIa bacteriocins. A multiple sequence alignment analysis of IIC and IID proteins revealed three sequence regions (two in IIC and one in IID) that distinguish members of the group from those of the other groups, suggesting that these amino acid regions confer the specific bacteriocin receptor function [136].

The receptor efficiencies of *Listeria*, *Enterococcus*, *Lactobacillus*, *Leuconostoc*, *Carnobacterium*, *Clostridium*, *Pediococcus* and *Streptococcus* varied in a pattern directly related to their phylogenetic position [136]. The species of *Enterococcus*, *Listeria* and *Carnobacterium* showed most active receptors and were highly sensitive to four IIa bacteriocins; the species of *Lactobacillus*, *Pediococcus* and *Clostridium* are also frequently inhibited by these bacteriocins, although they are often less sensitive; and the strains of *Streptococcus* and *Leuconostoc* are occasionally reported to be sensitive to class IIa bacteriocins at a low level. These results are in line with previous comparative analyses of the inhibitory spectra of class IIa bacteriocins [122,137]. Different strains of the same bacterial species can vary greatly in sensitivity to a given bacteriocin [122,138]. The variation in sensitivity might be due to differential expression levels of the receptor [136].

Generally, the conserved N-terminal region of class IIa bacteriocin was speculated to be involved in the receptor interaction, and the diverse *C*-terminal region was responsible for target cell species-specificity [136]. But some studies strongly suggest that the *C*-terminal region of class IIa bacteriocin might be involved in interaction between bacteriocin and its receptor [119,121,139,140]. Therefore it was speculated that *N*-terminal and *C*-terminal regions take part in the interaction with target cell receptor and that, they have different function during different stage of interaction. Synthesis of bacteriocin mutants and analogues provides valuable structure-activity relationships and tools to obtain further information on the peptide-receptor complex [117,119].

Resistance of *Listeria* spp. and other Gram-positive bacteria to class IIa bacteriocins was correlated with loss or reduction of expression of Man-PTS, inthe following phenotypes [132,135,141–143]: (i) absence of the IIAB subunit of Man-PTS in the proteomes of resistant bacteria [125,143]; (ii) mutations in the sigma transcription factor σ^54^ (*rpoN*) and the σ^54^-dependent transcription activator ManR of the *mpt* operon [124,126,127,144–146], (iii) a mutation in the promoter proximal *mptA* (IIA) cistron [125], and (iv) in-frame deletions in the *mptD* (IID) gene (which may have compromised the folding and stability of IID and IIC) [144]. Recently natural food isolates of *L. monocytogenes* with different susceptibilities to class IIa bacteriocins were investigated [135]. The results also identified Man-PTS as a key player in the mechanisms of resistance. At the same time, downregulation of the *mpoABCD* (mannose permease one) operon in *L. monocytogenes* was shown to promote resistance to class IIa bacteriocins [147]. The *mpoABCD* operon putatively encodes a PTS permease of the mannose family similar to that encoded by the mpt operon. *In silico* analysis indicated that *mpo* transcription might be dependent on σ^54^.

Bacterial strains sensitive to class IIa bacteriocins readily give rise to resistant mutants upon bacteriocin exposure. The development of highly tolerant and/or resistant strains may decrease the efficiency of bacteriocins as biopreservatives. The acquiring of resistance to bacteriocins can significantly affect physiological activity profile of bacteria, alter cell-envelope lipid composition, and also modify the antibiotic susceptibility/resistance profile of bacteria [148].

## 6. Discovery of Class IIa Bacteriocins

To date, traditional screening strategies have relied on detection of antimicrobial activity as the basis for discovery of new and potent bacteriocins [131]. New bacteriocins are detected and identified by screening large number of potential bacteriocin-producing bacteria for antimicrobial activity. The screened bacteriocins are then purified and characterized. These classic screening strategies are time-consuming and labor-intensive, so researchers need to explore and develop more rapid and higher-throughput approaches for identification of bacteriocins potential [149–152]. The PCR assays that target bacteriocin-coding genes or bacteriocin regulation-related genes for rapid detection of bacteriocins have been developed [152–156]. Most PCR assays can only detect known bacteriocins because they use specific primers which were designed according to previously characterized bacteriocins [154,155,157]. Więckowicz *et al.* have developed a rapid PCR assay with primers which were designed on the basis of a large scale alignment of class IIa bacteriocin genes. Several potentially novel bacteriocin-coding sequences were found by means of this high-throughput PCR assay [152].

A large number of LAB genomes have been published during the last decade [158,159]. At the same time, bioinformatics as well as new technologies such as transcriptomics, proteomics and metabolomic analysis have expanded tremendously in past decade. All of the above mentioned technologies have provided a basis for detection of bacteriocins by means of silico analysis [160]. Recently, there has been a trend from classical screening strategies for antimicrobial activity towards silico analysis of genomic data as computational approaches are able toaccelerate the process of novel antimicrobial peptides (AMPs) discovery and design [131,137,161,162].

Dirix *et al.* identified over 50 bacteriocins or bacteriocin-like peptides by screening for peptides containing a double-glycine leader sequence and the corresponding ABC transports in 165 fully sequenced bacterial genomes (including 45 Gram-positive bacteria and 120 Gram-negative bacteria) [161,162]. Diep *et al.* identified a new class IIa bacteriocin penocin A in the genome of *P. pentosaceus* ATCC 25745 by means of silico-based analysis. The antimicrobial activity of penocin A has been determined by experiments [137]. The silico analysis for prediction of bacteriocins, is a challenging task due to the small sizes and diversity in sequence, structure and function of bacteriocins [131].

Some databases and bioinformatics tools have been developed and designed for prediction of AMPs production by both Gram-positive and Gram-negative bacteria. For example, an antimicrobial peptide database (APD) was developed by means of sequence similarity and certain known principles of AMPs [163]. The database was updated in 2009 [164]. AMPer database provided hidden Markov models (HMMs) to automatically discover AMPs [165]. An integrated open-access database BACTIBASE (http://bactibase.pfba-lab-tun.org) [166], and a genome mining software BAGEL2 (http://bagel2.molgenrug.nl) [167] were specifically designed for AMPs discovery [168,169]. Wang *et al.* constructed a new method by means of sequence alignment and feature selection methods to predict AMPs [170]. Recently Fernandes *et al.* employed adaptive neuro-Fuzzy inference system (ANFIS) as a pattern recognition tool to classify a putative peptide as an AMP or non-AMP [171].

Quantitative structure–activity relationship (QSAR) modeling is one of the most broadly used chemoinformatics approaches. It can be defined as quantitative models that correlate the variation in measured biological activity with the variation in molecular structure among a series of chemical compounds. QSAR has been applied successfully to AMPs discovery [172–175]. The CAMEL database employed QSAR and artificial neural networks (ANN) to predict AMPs function [176]. Recently a novel quantitative prediction method of AMP was established by QSAR modeling based on the physicochemical properties of amino acids [177].

The activity of an AMP is commonly expressed as the threshold concentration (minimum inhibitory concentration, MIC) upon which bacterial growth is inhibited. Biophysical studies with model phospholipid membranes often identify concentration thresholds upon which the peptide behavior becomes disruptive through pore formation or membrane lysis [178–183]. The connections between *in vivo* MICs and thresholds in model membranes have been recently proposed [183,184]. Recently, Melo *et al.* developed an interaction model of antimicrobial peptides with biological membranes [178]. A straightforward and robust method was presented and used to implement this relationship. The methodology provides a basis for fast, cost-effective alternatives for screening AMPs, with potential application to high-throughput screening approaches. These tools will accelerate and optimize the discovery and identification of novel bacteriocins. Howerverthese bacteriocins still have to be verified by measuring their antimicrobial activities according to excepted experimental procedures.

## 7. Conclusions

A large number of new class IIa bacteriocins have been detected and purified in the last decade. Some class IIa bacteriocins with wide-spectrum antimicrobial activity have been reported and new discovery methods have been introduced. Acuña *et al.* presented a novel procedure for designing hybrid bacteriocins through fusion of microcins with class IIa bacteriocins in order to produce new wide-spectrum bacteriocins with high specific activity [185]. All of these advancements will accelerate the developments of class IIa bacteriocins.

## Supplementary Information

**Table S1:** Some characteristics of the class IIa bacteriocins.

Bacteriocin	Account Nucleotide	Account Protein	Prepeptie size (aa)	MP size (aa)	MP Mass (Da)	pI	Producer	Origin	References
**Group I**									
**Sub-group I-1**									
Avicin A	FJ851402.1	ACZ36002.1	61	43	4291.9	9.32	*E. avium* XA83	Feces of healthy infants	[ 21]
Bavaricin A/SppA	AF526262	AAM88858.1	61	43	4435.9	8.76	*L. sakei* MI401	Sourdough	[ 22]
Curvaticin L442 #		P84886.1					*L. curvatus* L442	Greek fermented sausage	[ 23]
Enterocin CRL35	AY398693	AAQ95741.1	58	43	4287	9.82	*E. mundtii* CRL35	Argentinian artisanal cheese	[ 24]
Enterocin HF		P86183		43	4333	9.37	*E. faecium* HS and TA29	Humans and fish	
Listeriocin 743A	AF330821.1	AAK19401.1	71	43	4484	9.98	*L. innocua* 743	Food	[ 186, 4]
Mundticin		P80925.1		43	4287		*E. mundtii* ATO6	Fresh chicory endive	[ 187]
Mundticin CRL35	AY444743	AAR26473.1	58	43	4287	9.82	*E. mundtii* CRL35/AT06	Artisanal cheese	[ 24]
Mundticin KS	AB066267	BAB88211.1	50	43	4287	9.82	*E. mundtii* NFRI 7393/AT06	Fresh chicory endive	[ 188]
Mundticin L	FJ899708.1	ACQ77507.1	58	43	4301.8	9.82	*E. mundtii* CUGF08	Alfalfa sprouts	[ 32]
Mundticin QU2				43 *	4287		*E. mundtii* QU 2	Fermented soybean	[ 189]
Pediocin ACCEL #							*P. pentosaceus* ACCEL		
Piscicocin CS526 #							*C. piscicola* CS526	Cold-smoked salmon	[ 190]
Piscicolin 126	AY812745	AAX21354.1	62	44	4417	9.32	*C.maltaromaticum* UAL26	Vacuum-packaged beef	[ 191]
Piscicolin 126	AF275938.1	AAK69419.1	62	44	4417	9.32	*C. piscicola* JG126	Spoiled ham	[ 192]
Piscicocin V1a				44	4417	9.32	*C. piscicola* V1	Fish	[ 193]
Sakacin P	DQ019413.1	AAY44078.1	61	43	4461.9	8.74	*L.curvatus* LTH1174	Meat fermentation	[ 38]
Sakacin P	DQ019414.1	AAY44080.1	61	43	4461.9	8.74	*L.curvatus* L442	Greek fermented sausage	[ 39]
Sakacin P	AY875983	AAW79057.1	61	43	4435.9	8.76	*L.sakei* I151	Sausage	[ 41]
Sakacin P	AF002276.1	AAB93970.1	61	43	4435.9	8.76	*L.sakei* LTH673	Meat fermentation	[ 40]
Sakacin P	NZ_AGBU01000084.1	ZP_09041901.1	61	43	4435.9	8.76	*L. curvatus* CRL 705	Fermented sausage	
Sakacin X	AY206863	AAP44569.1	61	43	4364	9.32	*L. sakei* 5	Malted barley	[ 101]
Sakacin X		ZP_09041912.1	61	43	4364	9.32	*L. curvatus* CRL 705	Fermented sausage	
**Sub-group I-2**									
Bifidocin B #				36	3801.5	8.05	*B. bifidum* NCFB 1454	Human isolate	[ 10, 11]
CoaA/Coagulin/CoaA	AF300457.1	AAG28763.1	62	44	4614.2	8.66	*B. coagulans* I _4_	Cattle feces	[ 194, 13]
Mutacin F-59.1		P86386.1		25 *			*S. mutans* 59.1		[ 9]
PapA	NC_004832.1	NP_857602.1	62	44	4627.2	8.66	*P. acidilactici* H		[ 195]
Pediocin	EU826148.1	ACF32966.1	62	44	4627.2	8.66	*P. acidilactici* MTCC 5101		
Pediocin A				44	4628	8.66	*P. pentosaceus* FBB61	Cucumber fementations	[ 51]
Pediocin AcH	S74PEDACH	AAA98337.1	62	44	4627.2	8.66	*P. acidilactici* H	Fermented sausage	[ 44]
Pediocin AcH				44	4627.2	8.66	*L. plantarum* WHE92	Soft cheese in France	[ 52]
Pediocin PA-1	HQ876214.1	AEH68223.1	62	44	4627.2	8.66	*E. faecium* Acr4		
Pediocin PA-1		AAB23877.1		44 *			*P. acidilactici*		[ 196]
Pediocin PA-1	M83924.1	AAA25559.1	62	44	4627.2	8.66	*P. acidilactici* PAC1.0.	Sorghum beer	[ 197, 42]
Pediocin PA-1				44	4628	8.66	*L. plantarum* DDEN 11007		[ 53, 66]
Pediocin PA-1				44	4628	8.66	*P. acidilactici* MM33	Human stool	[ 49]
Pediocin PP-1				44	4602.2	8.66	*P. pentosaceus* CBT8	Kimchi	[ 198]
Pediocin SJ-1							*P. acidilactici* SJ-1	Meat	[ 57]
Prepediocin AcH	S44537.1	AAC60413.2	62	44	4605.2	8.33	*P. acidila I ctici* Lb42-923		[ 44]
Prepediocin PA-1	AY705375.1	AAT95422.1	62	44	4627.2	8.66	*P. acidilactici* K10	Kimchi	[ 47]
**Sub-groupI-3**									
Leucocin C	LCCC_LEUME	P81053.2		43	4595	8.76	*L. mesenteroides* 6	Malted barley	[ 67]
Leucocin C-TA33a				36 *	4598		*L. mesenteroides* TA33a	Vacuum-packaged meat	[ 69]
Weissellin A				43	4450	9.32	*W. paramesenteroides* DX		[ 25]
**Sub-groupI-4**									
Bacteriocin 602		P86393.1		39	3864	7.2	*P. polymyxa* NRRLB-30509	Broiler chicken, crop	[ 26]
Bavaricin MN		P80493.2		42	4769	10.0	*L. sakei* MN	Meat	[ 27]
Divercin V41	AJ224003	CAA11804.1	66	43	4512.3	8.65	*C. divergens* V41	Fish viscera	[ 92, 199]
Divergicin M35		P84962.1		43	4518.75	8.3	*C. divergens* M35	Smoked salmon	[ 200]
Duracin GL	HQ696461.1	ADW93772.1	71	43	4966.7	8.74	*E. durans* 41D	Cheese product	
Enterocin A	X94181.1	CAA63890.1	65	47	4829	8.98	*E. faecium* CTC492	Fermented sausage	[ 91]
Enterocin A			65	47	4833	8.98	*E. faecium* WHE 81	Cheese	[ 201]
Enterocin A	NZ_GG692545.1	ZP_05660016.1	65	47	4831.6	8.98	*E. faecium* 1,230,933		
Enterocin A	AB038464.1	BAA92138.1	65	47	4831.6	8.98	*E. faecium* N15	Japanese rice-bran paste	[ 153]
Enterocin A/ EntA	AF099088.1	AAD29132	65	47	4831.6	8.98	*E. faecium* DPC1146		[ 202]
Enterocin BC25	AF240561.1	AAF44686.1	65	47	4831.6	8.98	*E. faecium* BC25		[ 203]
**Group II**									
Bacteriocin 31 /BacA	D78257.1	BAA11329.1	67	43	5007.8	9.72	*E. faecalis* YI717	Clinical sample	[ 72]
Bacteriocin 1580		P86394.1		35	3486	7.8	*B. circulans* NRRLB-30644	Broiler chicken, crop	[ 26]
Carnobacteriocin B2	L47121.1	AAB81310.1	66	48	4969.9	9.97	*C. piscicola* LV17B	Pork	[ 77, 108]
Bacteriocin 43	AB178871	BAF36626.1	74	44	5092.9	9.26	*E. faecium*		[ 204]
Bacteriocin RC714				43	4936.7	8.74	*E. faecium* RC714	Human fecal	[ 205]
Bacteriocin T8			74	44	5092.9	9.26	*E. faecium* T8	Children Infected with HIV	[ 206]
Enterocin SE-K4	AB092692.1	BAC20326.1	76	48	5356.2	9.93	*E. faecalis* K-4	Grass silage in Thailand	[ 207, 71]
Hiracin JM79	DQ664500	ABG47453.1	74	44	5092.9	9.26	*E. hirae* DCH5	Mallard ducks	[ 70]
Penocin A/PenA		YP_803635	60	42	4688.4	9.72	*P. pentosaceus* ATCC 25745		[ 137]
**Group III**									
**Sub-group III-1**									
Bacteriocin MC4-1	EU047916	ABW08100.1	71	43	4890.6	9.27	*E. faecalis* MC4		[ 34]
Carnocin CP52	CPU76763	AAB18989.1	66	48	4969.9	9.97	*C. piscicola* CP52	Cheese	[ 208]
Leucocin A	M64371.1/LEULAIP	AAA68003.1	61	37	3932.3	8.78	*L. gelidum* UAL 187	Vacuum-packaged meat	[ 209, 33]
Leucocin B-Ta11a	S72922.1	AAC60488.1	61	37	3931.6	8.78	*L. carnosum* Ta11a	Vacuum-packaged meat	[ 33]
Mesentericin 52A	AY286003	AAP37395.1	61	37	3869.5	8.78	*L. mesenteroides* subsp *. mesenteroides* FR52	Raw milk	[ 102]
Mesentericin Y105	X81803.1	*C*AA57405.1	61	37	3869.5	8.78	*L.mesenteroides* Y105	Goat's milk in France	[ 103]
Plantaricin 423	AF304384	AAL09346.1	56	37	3934.6	8.67	*L. plantarum* 423	Sorghum beer	[ 73, 210– 212]
Plantaricin C19				36	3845.3	9.88	*L. plantarum* C19	Fermented cucumbers	[ 213, 214]
									
Prebacteriocin SkgA2		ZP_08080540.1	56	38	4159.8	9.03	*L. ruminis* ATCC 25644	Human gastrointestinal tract	
Sakacin G	AF395533.1	AAM73712.1	55	37	3837.4	7.96	*L. sakei* 2512	Rhodia food collection	[ 105]
Sakacin G	FJ621568.1	ACM68469.1	55	37	3837.4	7.96	*L. sakei* R1333	Smoked salmon	[ 107]
Sakacin G	EU570253	ACB72724.1	55	37	3837.4	7.96	*L. sakei* CWBI-B1365	Raw poultry meat	[ 106]
Sakacin G	EU570253	ACB72725.1	55	37	3837.4	7.96	*L. sakei* CWBI-B1365	Raw poultry meat	[ 106]
**Sub-group III-2**									
Lactococcin MMFII		P83002.1		37	4144.6	7.25	*L. lactis* MMFII	Tunisian cheese	[ 76]
Bacteriocin		P86291.1		41	4601.3	7.25	*Lactococcus* sp.		
**Group IV**									
Carnobacteriocin BM1	L29058.1	AAA23014.1	61	43	4524.6	8.76	*C. piscicola* LV17B	Fresh pork	[ 77]
Curvacin A	S67323.1	AAB28845.1	59	41	4308.0	9.37	*L.curvatus* LTH 1174	Fermented sausage	[ 78]
Ubericin A	EF203953.1	ABQ23939.1	70	49	5270.5	9.35	*S. uberis* E		[ 8]
Enterocin P	GQ369522.1	ACU28817.1	71	44	4701.3	7.25	*E. faecium* IJ-31	Dairy products in Islamabad	[ 84]
Enterocin P	AF005726	AAC45870	71	44	4493	8.22	*E. faecium* P13	Spanish fermented sausage	[ 79]
Enterocin P	AY728265	AAU29394.1		44	4714.3	5.51	*E. faecium* GM-1	Feces of a newborn infant	[ 81]
Enterocin P-like	AY633748	AAT58220.1		44	4701.3	7.25	*E. faecium* ATB 197a		
Enterocin P-like	AB075741	BAC00780.1		40 *			*E. faecium* JCM5804T		[ 80]
Enterocin P	DQ867125	ABI29857.1		44	4629.3	8.22	*E. faecium* LHICA 51	Nonfermented animal foods	[ 82]
Enterocin P	DQ867124	ABI29856.1		44	4629.3	8.22	*E. faecium* LHICA 28-4	Nonfermented animal foods	[ 82]
Enterocin P	FJ416487	ACJ46053.1		44	4629.3	8.22	*E. faecium* LHICA 40-4	Nonfermented animal foods	[ 83]
Piscicocin V1b				43	4526	8.76	*C. piscicola* V1	Fish	[ 193]
Sakacin A	Z46867	CAA86942.1	59	41	4308.0	9.37	*L. sakei* Lb706	Meat	[ 215– 217]
**Group V**									
Bacteriocin E50-52		P85148.1		39	4124.9	8.12	*E. faecium* NRRL B-30746		[ 28]
**Group VI**									
Bacteriocin L-1077				37	3454	9.1	*L. salivarius* 1077	Healthy broiler chickens	[ 31]
**Group VII**									
Bacteriocin 37		P86395.1		30	3465.4	10.1	*P. polymyxa* NRRL B-30507	Broiler chicken, crop	[ 26]
**Group VIII**									
Acidocin A		BAA07120	81	58	6501.5	10.93	*L. acidophilus* TK9201		[ 29]
Bacteriocin OR-7				54	6214	10.32	*L. salivarius* NRRL B-30514	Cecal contents of chickens	[ 30]

aa, Amino acids; MP, Mature peptide;

# , the whole sequence of bacteriocin has not been determined, including Curvaticin L442 and bifidocin B;

* , some amino acids of bacteriocin has not been determined;

*B. circulans, Bacillus circulans; B. coagulans, Bacillus coagulans; B.bifidum, Bifidobacterium bifidum; C. divergens, Carnobacterium divergens; C. maltaromaticum, Carnobacterium maltaromaticum; C. piscicola, Carnobacterium piscicola; E. avium, Enterococcus avium; E. durans, Enterococcus durans; E. faecalis, Enterococcus faecalis; E. faecium, Enterococcus faecium; E. hirae, Enterococcus hirae; E. mundtii, Enterococcus mundtii; L. acidophilus, Lactobacillus acidophilus; L. carnosum, Leuconostoc carnosum; L. curvatus, Lactobacillus curvatus; L. gelidum, Leuconostoc gelidum; L. innocua, Listeria innocua; L. lactis, Lactococcus lactis; L. mesenteroides, Leuconostoc mesenteroides; L. pentosus, Lactobacillus pentosus; L. plantarum, Lactobacillus plantarum; L. ruminis, Lactobacillus ruminis; L. sakei, Lactobacillus sakei; L. salivarius, Lactobacillus salivarius; P. acidilactici, Pediococcus acidilactici; P. parvulus, Pediococcus parvulus; P. pentosaceus, Pediococcus pentosaceus; P. polymyxa, Paenibacillus polymyxa; S. mutans, Streptococcus mutans; S. uberis, Streptococcus uberis; W. paramesenteroides, Weissella paramesenteroides*; HIV, Human Immunodeficiency Virus.
